# Prevalence of abnormal findings on brain magnetic resonance (MR) examinations in adult participants of brain docking

**DOI:** 10.1186/1471-2377-5-18

**Published:** 2005-10-05

**Authors:** Yoshito Tsushima, Ayako Taketomi-Takahashi, Keigo Endo

**Affiliations:** 1 Department of Radiology, Motojima General Hospital, Ohta, 373-0033 Japan; 2 Department of Diagnostic Radiology and Nuclear Medicine Gunma University Hospital, Maebashi, Japan

## Abstract

**Background:**

To determine the prevalence of abnormal findings on brain magnetic resonance (MR) examinations in adult participants of brain docking in order to assess its usefulness.

**Methods:**

We analyzed screening brain MR examinations for 1113 adults (age, 52.6+/-8.5 years; range, 22–84; 761 male and 352 female) performed during 6-year period from April 1998 to March 2004. All participants voluntarily sought a brain MR examination at their own expense. All subjects were studied using the same 1.0-T MR scanner, on axial T1-weighted spin echo (SE) images, proton-density-weighted and T2-weighted fast SE images, and intracranial MR angiography (MRA). All abnormal findings were classified into three basic categories: (1) findings with no referral necessary; (2) findings not requiring further evaluation, but which needed to be reported to the referring physician; (3) findings requiring further evaluation.

**Results:**

Participants with abnormal MR findings requiring further evaluation accounted for 1.3 %, but five of seven suspected intracranial aneurysms were not confirmed by other imaging modalities (false positive). No malignant tumors or other life-threatening pathology was detected, and only three participants (0.27 %) with abnormalities underwent surgical treatment. No participant groups were identified from our data as being high risk for MR abnormal findings requiring further evaluation.

**Conclusion:**

Brain-docking participants had a variety of abnormalities on brain MR examinations, but only a small percentage of these findings required further evaluation. The usefulness of the brain docking with MRI and MRA has yet to be proven, and at this time we cannot approve this screening procedure.

## Background

"Brain docking", a method of screening for brain disease, has become popular in Japan in recent years. This unique Japanese practice, which may be performed as a part of an annual medical check-up, usually consists of brain magnetic resonance (MR) imaging and MR angiography (MRA) in addition to routine physical and laboratory examinations. It has been believed that brain docking may be beneficial for early diagnosis of some brain disorders, since it is well established that unexpected abnormalities are sometimes detected on brain MR examinations, usually in the setting of an investigation for a reason unrelated to the abnoramlity [1-4].

A screening examination is an examination on individuals who are at risk for a particular disease or condition, but who lack any signs or symptoms of the disease or condition, to determine if the disease or condition is present. It should be done only when clinical studies have demonstrated that screening examinations may do more good than harm [5]. The extraordinary spatial resolution of MR imaging seems to promise earlier intracranial disease detection and improved patient outcomes. However, to our knowledge, there has not been any scientific evidence to demonstrate that a screening brain MR examination may provide more benefit than harm to people being screened. Screening studies may raise issues regarding false-positive findings, overdiagnosis, and unnecessary additional medical examinations when the result is falsely interpreted as abnormal. Cost-effectiveness should also be determined.

In the current study, we analyzed the results of screening brain MR examinations in order to assess its usefulness. We also attempted to identify participant groups which are high risk for intracranial abnormalities on brain MR examinations, as limiting the screened population to those who are high risk for target disease would be better for cost effectiveness.

## Methods

### Participants and clinical data

We included 1113 consecutive adult participants (age, 52.6+/-8.5 years; range, 22–84; 761 male and 352 female) on whom brain MR examinations were performed during 6-year period from April 1998 to March 2004 as a part of an annual medical check-up in our hospital. All participants voluntarily sought a brain MR examination at their own expense. They usually believed that they were neurologically healthy at the time of the medical check-up, although some participants had a past history of brain infarction, bleeding, trauma or tumor. Some of them had recurrent headache or vertigo/dizziness, but they did not feel that immediate medical advice was required.

All participants underwent a clinical interview, physical and neurological examinations by a well-experienced neurologist. The presence of recurrent or chronic headache and vertigo/dizziness was always questioned by the neurologist. Since vertigo and dizziness were not always clearly differentiated, these two symptoms were combined in our analyses. Participants' demographic data consisted of age and sex. The body-mass-index (BMI), calculated as weight/height^2 ^(kg/m^2^), was used as the index of relative weight. Self-reported data on cigarette smoking were used to classify subjects as nonsmokers, current smokers of 1 to 20 cigarettes per day (moderate smokers), and those smoking more than 20 cigarettes per day (heavy smokers). Regular alcohol consumption was recorded as grams of average absolute ethanol per day, and categorized as follows; nondrinkers, low alcohol intake (< 60 g/day; moderate drinkers), and high alcohol intakes (≥ 60 g/day; heavy drinkers). Individuals were considered to have systemic hypertension if their blood pressure readings had repeatedly exceeded 140 mmHg systolic or 90 mmHg diastolic, if they were currently taking antihypertensive therapy, or if they had a past medical history of systemic hypertension. Diabetes mellitus was diagnosed by a fasting serum glucose concentration of > 140 mg/dl or under current treatment for diabetes. High serum cholesterol level was diagnosed by a fasting serum total cholesterol level of > 250 mg/dl, or having a past medical history of high serum cholesterol level.

Brain stroke was defined as a history of physician-diagnosed symptomatic infarction and hemorrhage. Transient ischemic attack (TIA) was not considered a brain stroke. Cardiac diseases were defined as a history of congestive heart failure, myocardial infarction, angina pectoris, left ventricular hypertrophy, or atrial fibrillation; or electrocardiographic evidence of past myocardial infarction, left ventricular hypertrophy, or atrial fibrillation.

### MR imaging and interpretation

All participants were studied on the same 1.0-T MR scanner (Magnex ^®^, Shimadzu, Kyoto, Japan), including axial T1-weighted spin echo (SE; TR = 450 ms, TE = 15 ms), and proton-density-weighted and T2-weighted fast SE (FSE; TR = 4000 ms, TE = 20 and 100 ms, echo train length = 8) images. The matrix was 256 * 192 and section thickness was 5 mm with a gap of 2.5 mm for all sequences. MR angiography (MRA) was also performed using time-of-flight (TOF) technique (TR = 40 ms, TE = 9 ms, flip angle = 20°, field of view = 200 mm; slice thickness = 1.0 mm; volume thickness = 54 mm; matrix = 256 * 174; number of acquisitions = 1; acquisition time = 6 minutes 15 seconds), and 16 projections of the MRA of the circle of Willis were created by a maximum-intensity projection (MIP) algorithm around the head-to-foot axis and right-to-left axis. Contrast-enhanced T1-weighted images were not obtained in any cases.

All MR images were interpreted by a board-certified diagnostic radiologist (YT) with 17-years of experience as a general radiologist, and all MR abnormalities were classified into three basic categories: (1) findings with no referral necessary; (2) findings not requiring further evaluation, but which needed to be reported to the referring physician; (3) findings requiring further evaluation. A well-experienced neurosurgeon also separately interpreted all images. When there was disagreement in image interpretation and neutral consensus could not be reached, the radiologist made the final decision. For MRA image interpretation, only the MIP images were evaluated, and the source images were not used.

White-matter signal abnormalities on MR images were considered present if visible as high intensity on proton-density and T2-weighted images, without prominent low intensity on T1-weighted images. According to Fazekas scale [6], periventricular hyperintensity (PVH) was graded as 0 = absence, 1 = caps or pencil-thin lining, 2 = smooth halo, and 3 = irregular PVH extending into the deep white matter. Separate deep white matter high intensities (DWMH) were rated as 0 = absent, 1 = punctuate foci, 2 = beginning confluence of foci, and 3 = large confluent areas. Both PVH and DWMH were considered abnormal when the grades were 2 or 3 [6], and classified as a finding not requiring further evaluation, but which needed to be reported to the referring physician.

When an intracranial aneurysm was suspected on MRA, a three-dimensional computed tomography (3D-CT) examination with contrast material or digital subtraction angiography (DSA) was recommended to confirm the diagnosis. 3D-CT was performed using a single-detector helical CT scanner (HiSpeed ^®^, GE-Yokogawa, Tokyo, Japan) with an intravenous bolus injection (2.5 ml/sec) of contrast material (iopamidol, Iopamiron 370 ^®^, Nihon Schering, Osaka, Japan; 80 ml), and surface rendering (SR) images were constructed from 40 or 50 axial images (thickness = 1.0 mm, pitch = 1.0) using an Advantage Workstation ^® ^(Version 3.1; GE-Yokogawa, Tokyo, Japan).

In our hospital, no approval of the ethics committee was necessary for this kind of a retrospective study. The Declaration of Helsinki principles was followed.

### Statistical analyses

The data were expressed by mean +/- standard deviation (SD). For statistical analyses, Student t test and Fisher exact test were used. P values less than 0.05 were considered significant. We set the screening cost for MR examination at US$200, although we know that the cost is much higher in US and EU countries.

## Results

Nine of 1113 participants had a past history of intracranial disease or trauma (two with cerebral bleedings, two with infarctions, two with brain contusions, one subdural hematoma [postoperative], one acoustic neurinoma [postoperative] and one intracranial aneurysm [operated]). Of 1113 participants, 939 (84.4 %) were categorized was having no abnormal findings or findings with no referral necessary. Abnormal MR findings were demonstrated in 15.6 % of the participants and were classified as follows: 159 (14.3 %) with findings not requiring further evaluation, but needed to be reported to the referring physician; 15 (1.3 %) with findings requiring further evaluation (Table 1,2). No malignant tumors or other life-threatening pathology were detected.

**Table 1:** Findings requiring further evaluation (15 participants, 1.3%).

**MR diagnosis**	**No. of cases**
Aneurysm, confirmed	1
Aneurysm, unconfirmed	6
Arachinoid cyst in the quadrigerminal plate cistern	1
Pituitary adenoma	3
Meningioma	1
Epidermoid tumor	1
Superficial siderosis	1
Major vessel stenosis	1

**Total**	**15**

**Table 2:** Findings not requiring further evaluation, but needing to be reported to the referring physician (159 participants, 14.3%).

**MR diagnosis**	**No. of cases**
White-matter signal abnormalities	113
Lacunar infarction	31
Old lobar or cerebellar infarction	13
Venous malformation	6
Arachinoid cyst in the middle cranial fossa	4
Old bleeding	4
Old traumatic lesion	2
Cerebral atrophy	2

**Total**	**175**

On MRA, seven intracranial aneurysms and one middle cerebral artery stenosis in eight participants were suspected (Table 1), and all were categorized as having findings requiring further evaluation. Of these eight participants, one aneurysm of the anterior communicating artery (8 mm) and a middle cerebral artery stenosis were confirmed by 3D-CT. Five presumed aneurysms, all of which were measured less than 5 mm in diameter on MRA, were not confirmed on 3D-CT (n = 2) or DSA (n = 3), thus we considered that the MRA findings of these five participants were to be false positives. These false positive findings were suspected to be due to the low image quality of the MRA images. One remaining participant did not wish to confirm the diagnosis of aneurysm on other imaging modalities.

In our screened population, three participants (0.27%) with abnormalities (one aneurysm, one pituitary adenoma and one epidermoid tumor) underwent surgical treatment.

Demographic and clinical data of the participants were summarized in Table 3. There were no statistically significant differences between the participants with and without abnormal MR findings requiring further evaluation.

The cost for a screening brain MR examination was approximately US$200 in Japan, thus the estimated cost for the identification of one participant with a finding requiring further evaluation (1.3 %) was US$ 14840. When the cases with unconfirmed intracranial aneurysms were excluded, there were nine participants who had findings requiring further evaluation, and the cost for the identification of one participant with a finding requiring further evaluation (0.8 %) rose to US$ 24733.

**Table 3:** Demographic and clinical data of the participants with and without abnormal MRI findings requiring further evaluation.

**Variables**	**Findings requiring further evaluation (n = 15)**			**No abnormality or findings not requiring further evaluation (n = 1098)**		
	
	**Age range**		**Total**	**Age range**		**Total**
				
	**34–59 y.o. (n = 11)**	**60–84 y.o. (n = 4)**		**34–59 y.o. (n = 910)**	**60–84 y.o. (n = 188)**	
**Age (y.o.)**			54.3 +/- 10.0			52.5 +/- 8.5
**(range)**			(34–75)			(22–84)*
**Sex, male:female**	9:2	1:3	10:5	622:288	129:59	751:347^§^
**BMI (kg/m-2)**	24.5 +/- 4.9	23.7 +/- 0.7	24.3 +/- 4.1	23.6 +/- 3.7	23.5 +/- 3.0	23.6 +/- 3.7
(range)	(19.5–35.6)	(22.7–24.4)	(19.5–35.6)	(13.8–59.0)	(13.8–33.5)	(13.8–59.0)*
**Hypertension**	3	1	4 (26.7%)	204 (22.4%)	59 (31.3%)	263 (24.0%)^§^
**Diabetes mellitus**	1	0	1 (6.7%)	94 (10.3%)	37 (19.7%)	131 (11.9%)^§^
**Hyperlipidemia**	6	1	7 (46.7%)	279 (30.7%)	74 (39.4%)	353 (32.0%)^§^
**Cardiac disease**	0	0	0 (0.0%)	21 (2.3%)	15 (8.0%)	36 (3.3%)^§^
**Heavy smoking**	1	0	1 (6.7%)	114 (12.5%)	16 (8.5%)	130 (11.8%)^§^
**Heavy drinker**	0	0	0 (0.0%)	40 (4.4%)	2 (1.1%)	42 (3.8%)^§^
**Headache**	1	0	1 (6.7%)	125 (13.7%)	9 (4.8%)	134 (12.2%)^§^
**Vertigo/Dizziness**	4	0	4 (26.7%)	111 (12.2%)	24 (12.8%)	135 (12.3%)^§^

There were no statistically significant differences between the participants with and without abnormal MR findings requiring further evaluation (*, Student t test; ^§^, Fisher exact test). No statistics were done on the breakdowns (age range) of each group.

## Discussion

In the current study, a variety of abnormal findings was discovered on screening brain MR examinations, but most of them were not serious. The prevalence of the abnormal findings requiring further evaluation in our study was only 1.3 %, and no malignant tumors or other life-threatening pathology was detected in any participants.

Some studies have attempted to determine the prevalence of abnormal findings in screening brain MR examinations, although most of these prior reports have focused on some specific abnormalities, such as silent infarctions [7], brain tumors [8] and unruptured intracranial aneurysms [9]. For instance, Onizuka et al. [8] reviewed screening brain MR examinations of 4000 individuals (24–85 years; mean, 56.0) without neurological signs or symptoms. They have focused on the prevalence of brain tumors, and found 11 incidental brain tumors (0.28 %). Some studies (Table 4) reported the prevalence of various incidental findings on MR examinations in neurologically healthy individuals in the setting of an investigation for other reasons [1-4]. Katzmen et al. [2] enrolled 1000 volunteers (3–83 years; mean, 30.6) who participated as control subjects for various research protocols, and reported the prevalence of 1.1 % of routine referral and 0 % of immediate referral necessary. Kim et al. [3] retrospectively reviewed 225 MR examinations performed for various research purposes in neurologically healthy children (1 month -18 years; mean 11.2 years), and reported a single lesion (< 1 %) requiring urgent referral. The prevalence of abnormal findings requiring further evaluation in our study (1.3 %) was close to the results previously reported in the adult population [1,2,4]. A very small percentage of participants (three participants; 0.27%) with abnormal findings required surgery in our study, and Yue et al. [1] also reported a similar low prevalence for abnormalities requiring surgery (0.25%).

**Table 4:** The studies evaluating the frequency of clinically important abnormalities on head MRI in neurologically normal participants of research protocols.

**Study & Year**	**No. of participants ** **(male/female)**	**Mean age (range)**	**MRI results**			
			
			No referral*	**Routine referral**	**Urgent referral**	**Immediate referral**
**Yue NC, et al. (1997) (1)**	3672	> 65	3608 (98.3 %)		64 (1.7 %)	
**Katzman GL, et al. (1999) (2)**	1000 (546/454)	30.6 (3–83)	971 (97.1 %)	18 (1.8 %)	11 (1.1 %)	0 (0 %)
**Kim BS, et al. (2002) (3)**	225 (100/125)	11.2 (0–18)	206 (91.6 %)	17 (8 %)	1 (< 1 %)	0 (0 %)
**Illes J, et al. (2004) (14)**	151 (82/69)	47.1 (18–90)	141 (93.4 %)	7 (4.6 %)	3 (2.0 %)	0 (0 %)

SD: standard deviation.

* Including the participants without any abnormal findings.

It was quite difficult to determine the cost effectiveness of brain docking, since a variety of intracranial diseases were discovered. It would be better to calculate quality-adjusted life-years (QALYs) gained by this screening procedure but this was also difficult for the same reason. From our study, however, since no malignant tumors or other life-threatening diseases (except for an aneurysm) were discovered in any participants, the cost-effectiveness of this screening procedure would largely depend on the prevalence of intracranial aneurysms. However, the wisdom of searching for and treating small asymptomatic intracranial aneurysms has been widely questioned.

Yoshimoto et al. [10] reported that screening asymptomatic populations to identify and treat unruptured aneurysms would not be cost-effective assuming the incidence of unruptured aneurysm of 3.0 % in 50-year-old subjects and annual rupture rates of 0.02–0.005. In the current study, the prevalence of a possible intracranial aneurysm was only 0.62 % (seven of 1113 participants), and only one aneurysm was surgically treated, thus based on their study it was less likely that the screening brain MR examination was cost-effective in our population. Baba et al. [11] also studied the cost-effectiveness of screening for asymptomatic unruptured intracranial aneurysms and concluded that such a mass screening was not cost-effective unless the diagnostic accuracy of MRA was considerably increased or the annual rate of subarachinoid hemorrhage due to unruptured aneurysms was high (0.01 to 0.02 per year). The diagnostic accuracy of intracranial aneurysms affects the usefulness of screening MRA. It has been reported that MRA can depict intracranial aneurysms 5 mm or larger with good accuracy [12]. However, MRA is less useful for the identification of smaller aneurysms, and the accuracy in the detection of intracranial aneurysms depends on the field strength, i.e. high field strength confers higher accuracy [13]. The development of both hardware and software of the MR unit also contributes to the improvement in the accuracy of the detection of intracranial aneurysms. The field strength of our MR unit used in this study was 1.0 T and both hardware and software were relatively old, so many small aneurysms may have escaped a correct diagnosis on the MRA in the current study. For a better understanding of the false positive aneurysms it is better to make comparisons to newer MR technologies, although it is quite difficult to discuss this issue only from our current data. Further studies using newer MR equipments are encouraged, and we are now trying to evaluate MRA images in the brain docking in our hospital using new 1.5-T MR equipment.

Not only MRA but also brain MRI may miss some conditions, and a diagnosis of normal might be inaccurate. However, it was less likely that the field strength of the MR unit and the quality of hardware and software would significantly affect the accuracy of detecting clinically important intracranial abnormalities on MRI, since findings less conspicuous than those detected in our study were unlikely to be clinically important. The lack of clinical follow-up, which may result in the underestimation of false negative findings, was also a shortcoming of our study. Currently, the false negative rate of brain docking is unknown.

Another important concern was that the diagnoses of uncertain or unimportant findings may require additional invasive testing or elaborate follow-up, which may place the persons at risk for unexpected health consequences and lead them to unwarranted health expenditures, and will result in increased patient anxiety [14]. In fact in the current study, five of seven presumed intracranial aneurysms were not confirmed by other imaging modalities.

From the view point of cost-effectiveness, it is better to limit the screened population who are at high risk for the target diseases. However, we were not able to identify any groups which were high risk for having intracranial MR abnormalities requiring further evaluation.

## Conclusion

Brain-docking participants had a variety of abnormalities on brain MR examinations, but the percentage of these findings requiring further evaluation was small. The usefulness of the brain docking with MRI and MRA has yet to be proven, and at this time we cannot approve this screening procedure.

## Competing interests

The author(s) declare that they have no competing interests.

## Authors' contributions

YT participated in the design of the study and performed the statistical analysis. KE and ATT conceived of the study, and participated in its design and coordination and helped to draft the manuscript. All authors read and approved the final manuscript.

## Pre-publication history

The pre-publication history for this paper can be accessed here:


